# Global Ophthalmology Research Output Relative to Disease Burden and National Income, 2011–2021

**DOI:** 10.3390/vision10040061

**Published:** 2026-09-01

**Authors:** Siddharth Gandhi, Michael Balas, Rachel Curtis, Tianwei Ellen Zhou, Ya-Ping Jin, Peng Yan

**Affiliations:** 1 Faculty of Medicine, Queen’s University, Kingston, ON K7L 3J8, Canada; 2 Department of Ophthalmology and Vision Sciences, University of Toronto, Toronto, ON M5T 3A9, Canada; 3 Department of Ophthalmology, Queen’s University, Kingston, ON K7L 5G2, Canada; 4 Toronto Western Hospital, University Health Network, University of Toronto, Toronto, ON M5T 2S8, Canada; 5 Kensington Vision and Research Center, 340 College Street, Suite 600, Toronto, ON M5T 3A9, Canada

**Keywords:** bibliometrics, disability-adjusted life years, global health, health disparities, ophthalmology, research prioritization

## Abstract

Purpose: To characterize ophthalmology research output relative to disease burden and national economic capacity after accounting for population size. Methods: We analyzed Scopus publications, Global Burden of Disease disability-adjusted life years (DALYs), and World Bank economic and population data from 2011 to 2021. Negative binomial and Gamma models evaluated publication volume and positive citation-weighted impact, adjusting for DALYs, gross domestic product (GDP) per capita, population, disease category, and publication year. Results: Refractive disorders accounted for 45.4% of DALYs and 23.2% of publication volume; cataract, 44.7% and 29.8%; glaucoma, 5.2% and 30.6%; and age-related macular degeneration, 3.8% and 15.2%. High-income countries accounted for 11.8% of burden, 67.2% of publication volume, and 74.2% of citation-weighted impact; lower-middle-income countries accounted for 49.1%, 7.9%, and 6.8%, respectively. DALYs were not independently associated with publication volume (adjusted count ratio [aCR], 0.99; 95% confidence interval [CI], 0.98–1.01; *p* = 0.278), whereas GDP per capita (aCR, 4.47) and population (aCR, 13.99) were strongly associated (both *p* < 0.001). For positive citation-weighted impact, DALYs had a small association (adjusted ratio of means [aRM], 1.05), whereas GDP per capita (aRM, 2.86) and population (aRM, 4.96) were strongly associated (all *p* < 0.001). Conclusions: After accounting for population size, absolute disease burden was not independently associated with publication volume, whereas national economic capacity remained strongly associated with both outcomes. These patterns should inform research and service-delivery priorities.

## 1. Introduction

Vision impairment remains a major global health challenge with substantial and persistent geographic inequalities [1,2,3,4,5,6,7,8]. A recent analysis of Global Burden of Disease 2021 data reported that the global age-standardized prevalence of blindness and vision loss increased from 12,454 per 100,000 population in 1990 to 15,784 per 100,000 in 2021, with particularly high prevalence in lower-Socio-Demographic Index settings [8]. Importantly, much of the burden reflects not only the availability of effective interventions but also gaps in their delivery. In 2026, an analysis of population-based surveys from 68 countries demonstrated substantial remaining deficiencies in effective cataract surgical coverage despite cataract surgery being an established and highly cost-effective treatment [9]. These contemporary data underscore that the global eye-health challenge encompasses both unresolved disease burden and unequal access to effective care. Scientific research contributes to the prevention, diagnosis, and treatment of ophthalmic disease and informs the organization and delivery of eye care services [10,11]. Because research funding, infrastructure, and expertise are finite, understanding how research activity corresponds to population health needs is important for research priority setting [12,13,14].

Health research priority-setting frameworks provide a broader basis for interpreting differences between disease burden and research activity. Because research resources are finite, priority setting involves choices among competing questions rather than allocation according to disease burden alone. Established frameworks emphasize transparent consideration of public health need, the importance of remaining evidence gaps, expected health benefit, feasibility, resource requirements, implementation context, and stakeholder priorities [15]. More recent ethical guidance further emphasizes that research priority setting has an equity dimension because decisions about which questions and populations receive research investment influence how the potential benefits of research are distributed; fairness, inclusion, and whose interests are represented are therefore integral considerations [16]. The nature of the unanswered question is also important: research directed toward discovering or evaluating interventions addresses a different need from implementation and health-systems research examining how proven interventions can be delivered safely, effectively, affordably, and equitably in routine practice [17]. Research activity may also be shaped by national economic capacity, academic infrastructure, commercial incentives, technological feasibility, and the availability of funding [18,19,20,21]. Accordingly, differences between disease burden and publication output should not be interpreted against a simple expectation of proportionality, but rather within the broader context of scientific uncertainty, health-system needs, equity, and the opportunity costs of finite research resources.

This study examined global ophthalmology research output relative to disease burden for five major causes of vision impairment: age-related macular degeneration (AMD), cataract, glaucoma, refractive disorders, and trachoma. By integrating publication, disease-burden, economic, and population data from 2011 to 2021, we aimed to characterize differences in publication volume and citation-weighted impact across diseases, countries, and income groups and to determine whether disease burden and national economic capacity were independently associated with these bibliometric outcomes after accounting for national population size.

## 2. Methods

### 2.1. Study Design and Data Sources

This cross-sectional study assessed global ophthalmology research activity relative to disease burden from 1 January 2011 to 31 December 2021. The analysis integrated publicly available disease-burden, economic, and population data with bibliographic metadata obtained from Scopus.

Disease-burden data were obtained from the Global Burden of Disease database [22]. We extracted annual country- and territory-specific estimates of disability-adjusted life years (DALYs) from 2011 to 2021 for five causes of vision impairment: AMD, cataract, glaucoma, refractive disorders, and trachoma. These were the vision-related causes for which consistently available, disaggregated DALY estimates could be matched to disease-specific publication searches. Retinoblastoma was excluded because it is a rare pediatric malignancy with a research landscape that differs substantially from the other chronic and infectious causes of vision impairment examined.

Publication data were retrieved from Scopus using five separate disease-specific searches for AMD, cataract, glaucoma, refractive disorders, and trachoma. Each search applied disease-specific terms to the title, abstract, author keyword, and Scopus indexed-term fields. Searches were restricted to journal articles published from 2011 to 2021, records indexed with the exact keyword “Human,” and journal source types. The complete search strategies are provided in Supplement S1. Each retrieved record was assigned to the disease category corresponding to the search that identified it. Records retrieved by more than one disease-specific search were retained once in each applicable disease category.

Economic and population data were obtained from the World Bank [23]. We extracted annual gross domestic product (GDP) per capita in current United States dollars, annual World Bank income classifications, categorized as high income, upper-middle income, lower-middle income, or low income, and annual national population. Population was obtained from the World Bank Population, Total series (indicator SP.POP.TOTL). For Taiwan, which was not included in the World Bank dataset, corresponding economic data were obtained from the International Monetary Fund [24]. Primary disease-burden, publication, and economic data were extracted between 1 July and 31 July 2025. Population data were retrieved from the World Bank in August 2026.

Under University of Toronto institutional guidance, Research Ethics Board review was not required because the study relied exclusively on publicly available aggregate data and bibliographic metadata and did not involve identifiable private information.

### 2.2. Data Processing and Variable Definitions

The five disease-specific Scopus searches initially identified 83,491 publication records. Records were processed separately within each disease category, and duplicate records within the same disease category were identified using the Scopus electronic identifier and retained once. After within-disease deduplication, 79,124 article–disease records remained. Because an article could address more than one included disease, records retrieved by multiple disease-specific searches were retained once within each applicable disease category.

To assign a single country of origin to each article, a publication was attributed to the country of the corresponding author’s primary affiliation, representing institutional origin rather than study location, whenever this information was available. When corresponding-author affiliation information was unavailable, a publication was attributed to a country only when all listed authors had affiliations from the same single country and no affiliation from another country was present. Publications that did not meet either criterion were excluded from country-level analyses. Country names were standardized across the publication, disease-burden, economic, and population datasets.

A total of 2187 article–disease records lacked an assignable country and were excluded. The country-attributed dataset therefore comprised 76,937 article–disease records, representing 64,671 globally unique Scopus-indexed articles and publications from 148 countries. The distinction between article–disease records and unique articles reflects the retention of articles retrieved in more than one disease-specific search within each applicable disease category.

Two primary measures of research output were defined: publication volume and citation-weighted impact. Publication volume was defined as the number of article–disease records assigned to each country, disease, and year. For longitudinal statistical models, publication volume was measured annually, whereas country- and disease-level descriptive summaries used cumulative publication counts over the 2011–2021 study period.

Citation-weighted impact was calculated for each article as log_10_(1 + citation count). The logarithmic transformation reduced the influence of a small number of highly cited articles while retaining information on citation performance. Citation-weighted impact for each country, disease, year, or other analytic stratum was calculated by summing the transformed article-level scores. Citation counts reflected values available in Scopus at the time of data extraction and were not normalized by publication year, journal, or field.

Publication data were merged with disease-burden data by standardized country or territory, disease category, and year. Annual GDP per capita, World Bank income classification, and national population were then assigned by country and year. Country–disease-year observations with no identified publications were retained with publication volume and citation-weighted impact coded as zero when corresponding disease-burden and economic data were available.

To compare research output relative to disease burden, we calculated the Research-to-Burden Ratio (RBR), defined as cumulative publication volume divided by cumulative DALYs [12]. Higher RBR values indicated greater publication output per unit of disease burden. The Impact-to-Burden Ratio (IBR) was defined as cumulative citation-weighted impact divided by cumulative DALYs and represented citation-weighted research impact per unit of disease burden. RBR and IBR were used as descriptive bibliometric measures and were not intended to represent healthcare access, quality of care, health-system performance, or an optimal level of research investment.

For analyses requiring a single representative income classification per country, each country was assigned its modal, or most frequently occurring, World Bank income group over the 2011–2021 study period. Modal income classifications were used for descriptive comparisons among high-income, upper-middle-income, lower-middle-income, and low-income countries; comparisons between high-income countries and low- and middle-income countries; and the identification of high-burden, low-publication country–disease pairs.

### 2.3. Statistical Analysis

All statistical analyses were performed using Python 3.12.4. All tests were two-sided, and *p* < 0.05 was considered statistically significant. Descriptive analyses summarized publication volume, citation-weighted impact, disease burden, the RBR, and the IBR by country, disease category, and national income group.

To evaluate the country-level association between disease burden and research output, cumulative DALYs, publication volume, and citation-weighted impact were summed across diseases and years for each country or territory. Spearman rank correlation coefficients were calculated between cumulative DALYs and publication volume and between cumulative DALYs and citation-weighted impact. Ninety-five percent confidence intervals (CIs) were calculated using the Fisher z approximation for each correlation coefficient. To identify factors associated with research output, two multivariable regression models with log links were developed. The first evaluated annual publication volume using a negative binomial distribution because publication counts are non-negative integer data that exhibit overdispersion. The second evaluated positive annual citation-weighted impact using a Gamma distribution because positive outcome values were continuous and right-skewed. Observations with zero citation-weighted impact were excluded; accordingly, this model estimated associations with the magnitude of citation-weighted impact conditional on positive impact. Both models were fitted using heteroskedasticity-robust HC0 standard errors.

Both primary models included annual DALYs, GDP per capita, annual national population, disease category, and publication year as predictor variables. Population was natural-log transformed because of its right-skewed distribution. DALYs, GDP per capita, and log population were standardized to have a mean of zero and a standard deviation of one to facilitate comparison of effect estimates. Disease category and publication year were included as categorical variables, with AMD and 2011 serving as the reference categories, respectively. Regression analyses were restricted to observations with complete model covariates. Before population linkage, 10,880 country–disease-year observations were regression-complete. World Bank population data were unavailable for Taiwan, excluding 55 additional observations and leaving 10,825 observations for the publication-volume model. The citation-weighted-impact model included 3036 observations with positive citation-weighted impact; 7789 observations with zero citation-weighted impact were excluded from the Gamma model. Potential multicollinearity among DALYs, GDP per capita, and log population was assessed using variance inflation factors.

Model coefficients are reported as adjusted count ratios (aCRs) for the publication-volume model, representing multiplicative ratios of expected annual publication counts, and adjusted ratios of means (aRMs) for the citation-weighted-impact model, obtained by exponentiating the regression coefficients. No exposure offset was used in the negative binomial model. As a sensitivity analysis, both models were refitted on the same population-complete sample after omitting population to assess the influence of accounting for country size on the estimated associations of DALYs and GDP per capita with research output.

To identify country–disease pairs with high measured disease burden and low bibliometric output relative to countries with similar economic capacity, cumulative DALYs and publication volume were calculated for each country–disease pair. Countries were grouped according to their modal World Bank income classification over the study period. A high-burden, low-publication country–disease pair was defined as one in which cumulative disease burden ranked in the top quartile (≥75th percentile) within its modal income group, while cumulative publication volume for the same disease ranked in the bottom quartile (≤25th percentile) within that income group. This descriptive designation was not intended to establish that additional research was necessarily preferable to investment in implementation or service delivery.

## 3. Results

### 3.1. The Global Landscape of Research and Disease Burden

The five disease-specific searches yielded 76,937 country-attributed article–disease records after within-disease deduplication and country attribution, representing 64,671 globally unique Scopus-indexed articles from 148 publication countries. After integration with disease-burden and economic data, the analytic dataset included 11,220 country–disease-year observations across 204 countries and territories from 2011 to 2021.

**Figure 1:**
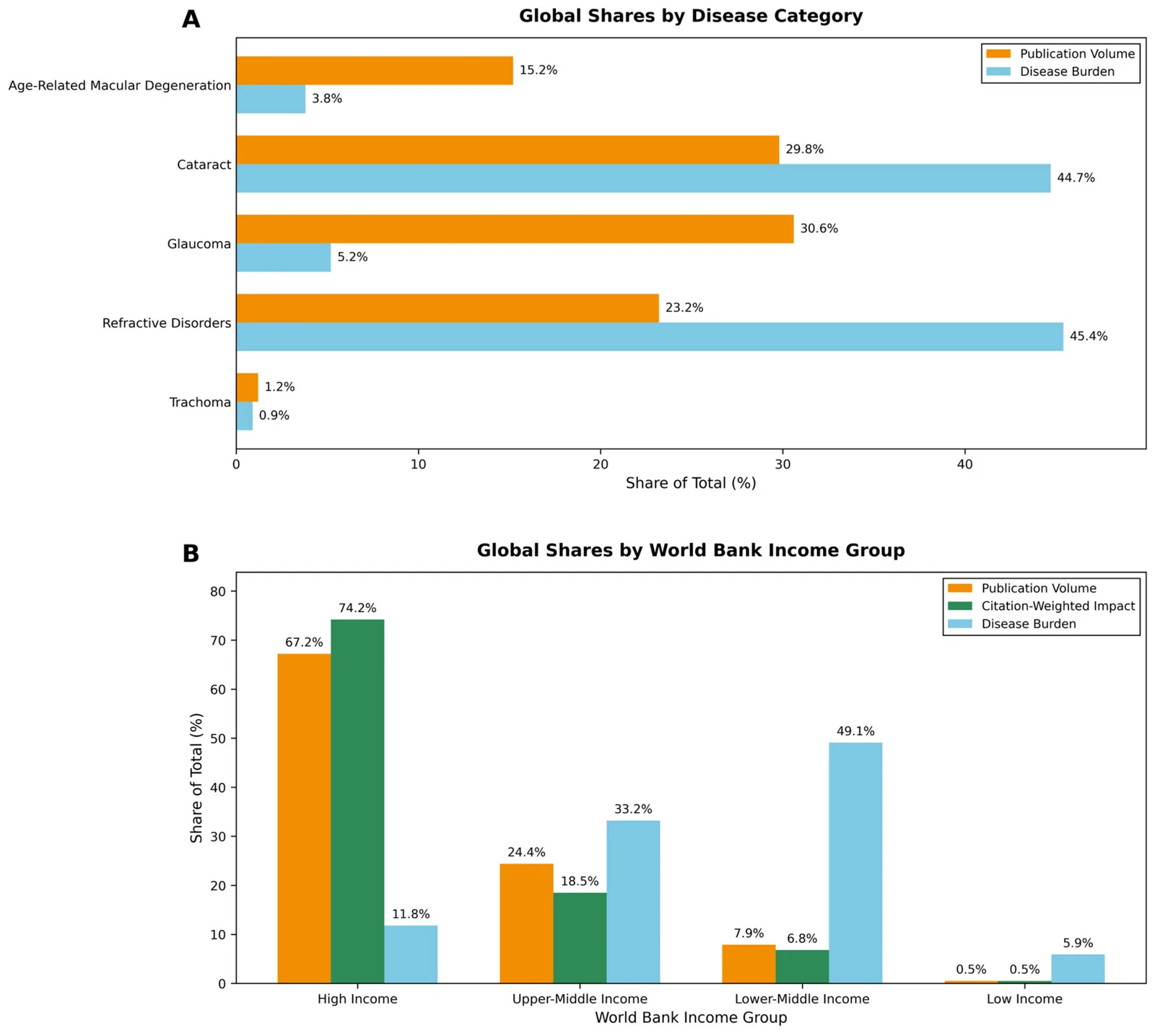
Global distribution of ophthalmology research output and disease burden by disease category and World Bank income group, 2011–2021. Panel (**A**) shows the percentage of total publication volume and cumulative disease burden attributable to age-related macular degeneration, cataract, glaucoma, refractive disorders, and trachoma. Panel (**B**) shows the percentage of global publication volume, citation-weighted impact, and cumulative disease burden attributable to each country’s modal World Bank income group during the study period. Publication volume was defined as the number of country-attributed article–disease records. Citation-weighted impact was calculated by summing log_10_(1 + citation count) across article–disease records. Disease burden was measured using cumulative disability-adjusted life years (DALYs). One country-attributed article–disease record lacking an available income-group classification was excluded from the income-group summary in Panel (**B**). The figure was generated using Python 3.12.4 with Matplotlib (version 3.11.1).

The distribution of research output differed substantially from the distribution of disease burden across the five disease categories (Figure 1A). Refractive disorders accounted for 45.4% of cumulative DALYs but 23.2% of publication volume, while cataract accounted for 44.7% of DALYs and 29.8% of publication volume. Together, refractive disorders and cataract accounted for 90.1% of cumulative disease burden but 53.0% of publication volume.

In contrast, glaucoma accounted for 5.2% of cumulative DALYs but 30.6% of publication volume, while AMD accounted for 3.8% of DALYs and 15.2% of publication volume. Together, glaucoma and AMD accounted for 9.0% of cumulative disease burden but 45.8% of publication volume. Trachoma represented the smallest share of both cumulative disease burden and publication volume, accounting for 0.9% and 1.2%, respectively.

The distribution of research output also differed across national income groups (Figure 1B and Supplement S2). High-income countries accounted for 11.8% of cumulative disease burden but produced 67.2% of publication volume and 74.2% of citation-weighted impact. Upper-middle-income countries accounted for 33.2% of disease burden, 24.4% of publication volume, and 18.5% of citation-weighted impact. Lower-middle-income countries bore 49.1% of cumulative disease burden but produced 7.9% of publication volume and 6.8% of citation-weighted impact. Low-income countries accounted for 5.9% of disease burden and 0.5% of both publication volume and citation-weighted impact.

Across low- and middle-income countries collectively, cataract and refractive disorders accounted for 91.2% of cumulative disease burden and 61.4% of publication volume. In high-income countries, refractive disorders accounted for the largest share of cumulative disease burden at 49.1% but represented 20.9% of publication volume.

### 3.2. Country-Level Patterns of Research Output Relative to Disease Burden

**Figure 2:**
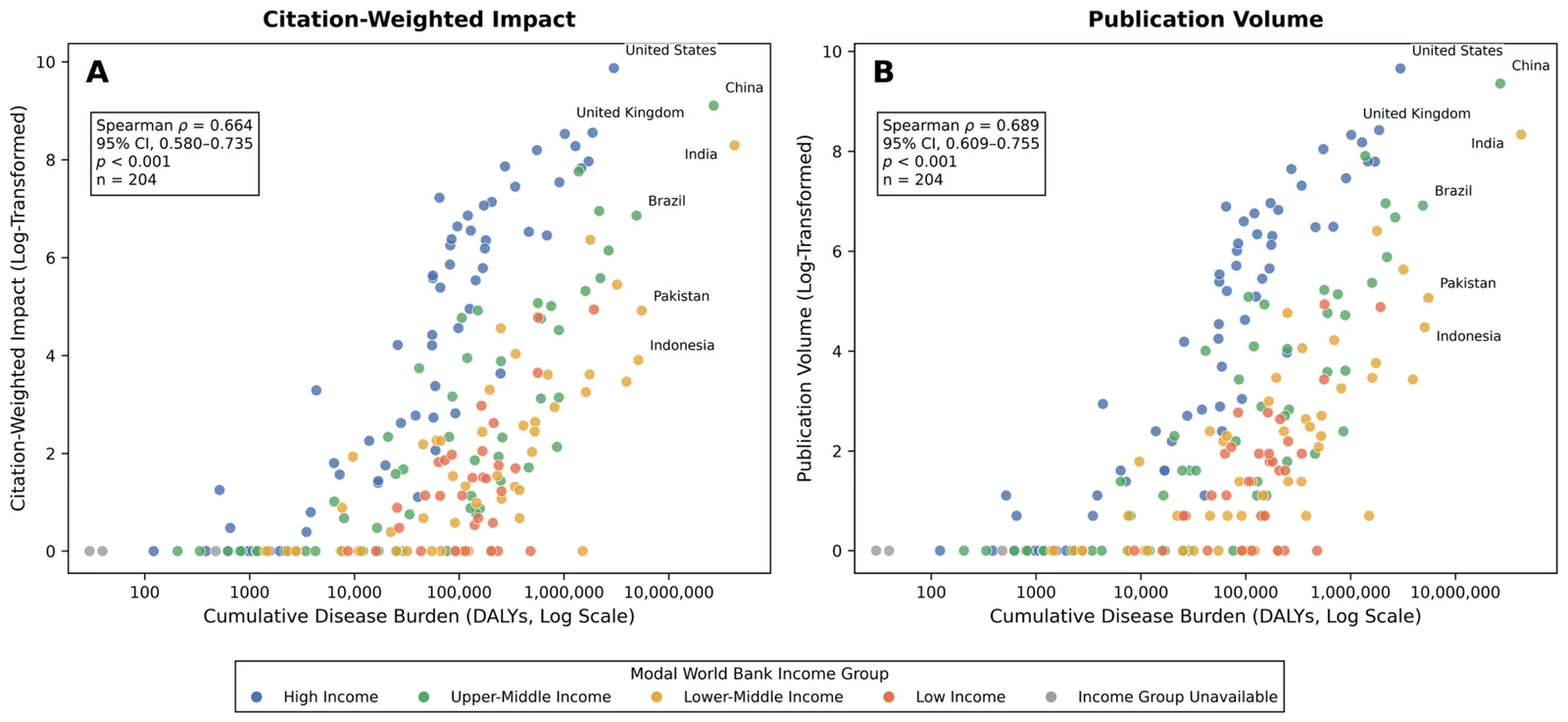
Unadjusted country-level associations between cumulative disease burden and ophthalmology research output, 2011–2021. Panel (**A**) shows the association between cumulative disease burden, measured in disability-adjusted life years (DALYs), and cumulative citation-weighted impact (Spearman ρ = 0.664; 95% CI, 0.580–0.735; *p* < 0.001). Panel (**B**) shows the association between cumulative disease burden and cumulative publication volume (Spearman ρ = 0.689; 95% CI, 0.609–0.755; *p* < 0.001). Publication volume was defined as the number of country-attributed article–disease records, and citation-weighted impact was calculated by summing log_10_(1 + citation count) across article–disease records. Cumulative DALYs are displayed on logarithmic x-axes. The y-axis outcomes are displayed as ln(1 + cumulative value), allowing countries and territories with zero publication volume or citation-weighted impact to remain visible. Each point represents one country or territory and is colored according to its modal World Bank income group during the study period. Selected countries are labeled for reference. The figure was generated using Python 3.12.4 with Matplotlib.

In unadjusted country-level analyses across 204 countries and territories, cumulative disease burden was positively correlated with citation-weighted impact (Figure 2A; Spearman ρ = 0.664; 95% CI, 0.580–0.735; *p* < 0.001) and publication volume (Figure 2B; Spearman ρ = 0.689; 95% CI, 0.609–0.755; *p* < 0.001). Although countries with greater disease burden generally produced more research in these unadjusted analyses, substantial variation in research output remained among countries with similar levels of burden.

The United States had the highest cumulative publication volume, with 15,620 article–disease records (Supplement S3), and the highest citation-weighted impact, with a cumulative score of 19,395 (Supplement S4). Research portfolios also differed among countries with high publication output. AMD represented approximately 20% of publication volume in the United States, the United Kingdom, and Germany. In contrast, refractive disorders and cataract represented 32.7% and 31.8%, respectively, of China’s publication volume, together accounting for 64.5% of its research portfolio. AMD represented 5.8% of publication volume in India.

**Figure 3:**
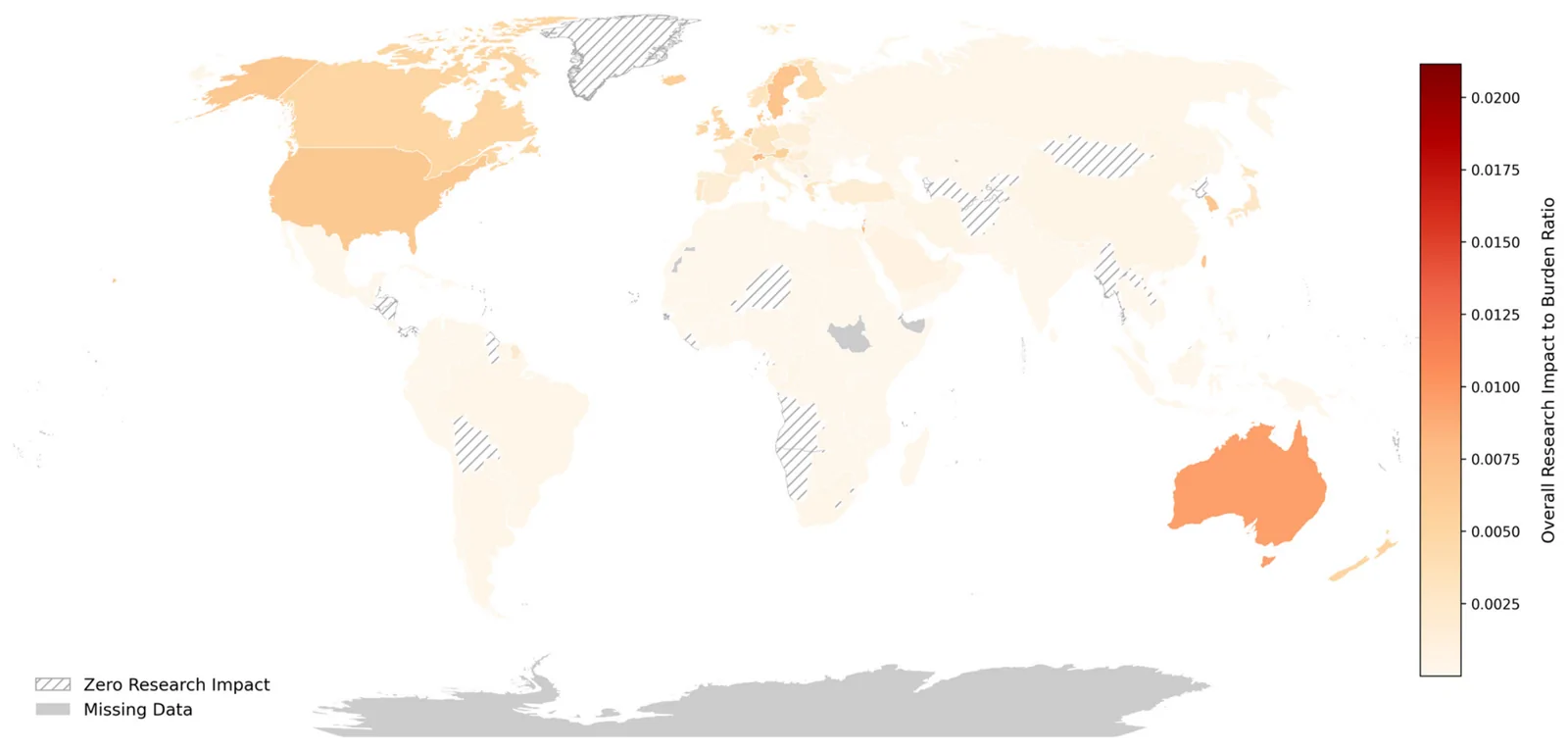
Geographic distribution of citation-weighted research impact relative to disease burden across five ophthalmic disease categories, 2011–2021. The map displays the overall Impact-to-Burden Ratio (IBR) for each country or territory, calculated as cumulative citation-weighted impact divided by cumulative disability-adjusted life years (DALYs), with both measures summed across age-related macular degeneration, cataract, glaucoma, refractive disorders, and trachoma. Citation-weighted impact was calculated by summing log_10_(1 + citation count) across country-attributed article–disease records. Darker shading indicates greater citation-weighted impact per unit of disease burden. Diagonal hatching indicates zero citation-weighted impact, and gray shading indicates missing data. The map was generated using Python 3.12.4 with GeoPandas (version 1.1.4) and Matplotlib.

The geographic distribution of citation-weighted research impact relative to disease burden, measured using the IBR, is shown in Figure 3. Higher IBR values indicate greater citation-weighted impact per unit of cumulative disease burden. Overall IBR values were highest in Singapore, Australia, and Israel. However, geographic patterns differed by disease (Supplements S5–S9). Australia had among the highest IBR values for refractive disorders and trachoma (Supplements S6 and S7), whereas higher glaucoma IBR values were distributed more broadly across high-income regions, including North America and Western Europe (Supplement S8).

RBRs also varied by income group and disease category (Table 1). Among high-income countries, glaucoma and AMD had RBRs of 95 and 68 article–disease records per 10,000 DALYs, respectively. The trachoma RBR in high-income countries was substantially higher at 1504 article–disease records per 10,000 DALYs, reflecting the very low cumulative trachoma burden in this income group.

Lower-middle-income countries had comparatively low RBRs for conditions accounting for large shares of disease burden. Cataract accounted for more than 37 million cumulative DALYs in lower-middle-income countries but had an RBR of approximately 1 article–disease record per 10,000 DALYs. The rounded RBR for refractive disorders was 0 per 10,000 DALYs in both lower-middle-income and low-income countries.

The prespecified analysis identified 34 high-burden, low-publication country–disease pairs, defined by disease burden in the top quartile and publication volume in the bottom quartile within the same disease and modal income-group stratum (Supplement S10). In each identified pair, the bottom-quartile publication threshold was zero, indicating that the corresponding country–disease pair had no identified publications in the dataset. These pairs occurred predominantly in low- and lower-middle-income countries but were not limited to these income groups. Peru, an upper-middle-income country, met the definition for AMD, with 36,595 cumulative DALYs and no corresponding article–disease records. The largest identified pair by cumulative burden was cataract in Myanmar, with 913,722 DALYs and no corresponding article–disease records. Because these percentile thresholds were defined within each disease and modal income-group stratum, “high burden” is relative to the comparison stratum rather than an absolute burden threshold. Consequently, pairs may be identified in strata where absolute disease burden is uniformly very low and should be interpreted as percentile-based screening indicators rather than substantive evidence of a large unmet research need.

### 3.3. Regression Analysis

In multivariable models including annual DALYs, GDP per capita, national population, disease category, and publication year, annual DALYs were not independently associated with publication volume (aCR, 0.99; 95% CI, 0.98–1.01; *p* = 0.278) (Table 2). In contrast, a 1-standard-deviation increase in GDP per capita was associated with greater expected publication volume (aCR, 4.47; 95% CI, 4.22–4.73; *p* < 0.001), as was a 1-standard-deviation increase in log population (aCR, 13.99; 95% CI, 13.02–15.03; *p* < 0.001). For positive citation-weighted impact, DALYs had a small positive association (aRM, 1.05; 95% CI, 1.03–1.06; *p* < 0.001), while GDP per capita (aRM, 2.86; 95% CI, 2.72–3.00; *p* < 0.001) and log population (aRM, 4.96; 95% CI, 4.63–5.32; *p* < 0.001) were more strongly associated.

Relative to AMD, glaucoma, cataract, and refractive disorders were each associated with higher publication volume and higher positive citation-weighted impact after adjustment for DALYs, GDP per capita, population, and publication year. The associations were largest for cataract publication volume (aCR, 2.54; 95% CI, 2.24–2.89; *p* < 0.001) and glaucoma citation-weighted impact (aRM, 1.55; 95% CI, 1.40–1.72; *p* < 0.001). Trachoma was associated with lower publication volume (aCR, 0.10; 95% CI, 0.08–0.12; *p* < 0.001) and positive citation-weighted impact (aRM, 0.25; 95% CI, 0.20–0.30; *p* < 0.001).

Relative to 2011, expected publication volume was higher in 2015 and in each year from 2016 through 2021, reaching an aCR of 1.69 in 2021 (95% CI, 1.38–2.08; *p* < 0.001). For positive citation-weighted impact, only 2016 differed significantly from 2011 (aRM, 1.23; 95% CI, 1.01–1.50; *p* < 0.05). In sensitivity models omitting population but using the same population-complete sample, the DALY association with publication volume was substantially larger (aCR, 27.68; 95% CI, 15.66–48.92; *p* < 0.001), supporting the importance of accounting for country population size (Supplement S11).

## 4. Discussion

This cross-sectional bibliometric analysis found that global ophthalmology research output was unevenly distributed relative to disease burden across disease categories, countries, and national income groups. Refractive disorders accounted for 45.4% of cumulative DALYs but 23.2% of publication volume, cataract accounted for 44.7% and 29.8%, glaucoma accounted for 5.2% and 30.6%, and AMD accounted for 3.8% and 15.2%, respectively. High-income countries accounted for 11.8% of measured disease burden but produced 67.2% of publication volume and 74.2% of citation-weighted impact. In the primary multivariable models accounting for national population, DALYs were not independently associated with publication volume, whereas GDP per capita and population were strongly associated with publication volume. DALYs retained only a small association with positive citation-weighted impact, while GDP per capita and population remained strongly associated with that outcome. Collectively, these findings indicate that national economic capacity and demographic scale are more strongly associated with ophthalmology research activity than absolute disease burden alone.

The relatively small 5.9% share of cumulative DALYs attributed to low-income countries also warrants clarification. This value represents the share of absolute cumulative DALYs across the five included disease categories rather than a per capita or age-standardized measure of disease burden. Accordingly, the income-group totals reflect population size and demographic structure in addition to disease frequency. This distinction is particularly relevant because several of the included conditions, including cataract, glaucoma, and AMD, are strongly age-associated, and population aging substantially influences the burden of vision loss [3]. The 5.9% value therefore should not be interpreted as indicating that individuals in low-income countries experience less vision-related disadvantage or as evidence of a monotonic relationship between national income and individual disease risk. In addition, the analysis relied on modeled Global Burden of Disease estimates and did not incorporate uncertainty intervals around DALYs; consequently, estimation uncertainty may also contribute to differences among income groups [22]. The income-group percentages should therefore be interpreted as the distribution of modeled absolute burden within the five conditions examined rather than as comparative population-level rates of vision impairment.

The income-group and country-level analyses describe different features of the data. The income-group analysis compares each group’s share of global disease burden with its share of global publication output, whereas the country-level correlations describe unadjusted associations between absolute burden and research output. Although countries with greater absolute disease burden generally produced more research in these unadjusted comparisons, the primary population-adjusted model showed no independent association between DALYs and publication volume. GDP per capita remained strongly associated with publication volume, indicating that national economic capacity is associated with scholarly output independent of country population size and measured disease burden. The marked difference between models with and without population adjustment suggests that country size contributed substantially to the apparent association between absolute DALYs and publication volume.

These findings are best interpreted within a health research priority-setting framework rather than a model in which publication output is expected to correspond directly to disease burden. Disease burden represents one consideration in prioritization, alongside the importance of unresolved questions, expected health benefit, feasibility, implementation needs, resource requirements, and stakeholder priorities [15]. An equity-oriented approach additionally considers who participates in setting research agendas and which populations are likely to benefit from the knowledge generated [16]. Importantly, high disease burden can arise from different underlying problems. In some settings, clinically important uncertainty remains regarding disease prognosis, prevention, diagnosis, or the effects of interventions, making additional basic or clinical research appropriate. In others, effective interventions are already established, and persistent burden reflects structural barriers involving financing, workforce, infrastructure, affordability, quality, or service delivery. Research may help identify and evaluate strategies to overcome such barriers through implementation and health-systems research [17,25], but research activity alone cannot supply the services, workforce, infrastructure, or financing required to eliminate them. Thus, differences between disease burden and bibliometric output do not by themselves establish research underinvestment or imply that additional publications are the appropriate response. Rather, they identify settings in which locally informed priority setting should distinguish unresolved knowledge questions from implementation problems and service-delivery deficits before determining whether the appropriate response is additional research, implementation activity, or direct investment in care.

Differences in research output across diseases may reflect variation in clinical characteristics, available treatments, scientific opportunities, and funding incentives. Glaucoma and AMD are chronic, age-associated causes of irreversible vision loss for which substantial basic and clinical research continues to address uncertainties in pathogenesis, prevention, diagnosis, and treatment. Cataract and refractive disorders differ in that effective interventions are already established, but this does not imply that important research questions have been exhausted. Rather, the nature of the relevant questions may shift from whether an intervention is efficacious toward how it can be delivered safely, effectively, affordably, and equitably. For cataract, implementation and health-systems research may address surgical coverage, workforce and facility capacity, referral pathways, affordability, uptake, postoperative follow-up, and variation in surgical quality and outcomes [17,25,26]. For refractive disorders, relevant questions include screening and case detection, affordability and availability of spectacles or other correction, treatment uptake and adherence, integration into school and primary-care systems, and effective refractive error coverage [17,25,27]. These forms of research are distinct from basic or therapeutic discovery research but may be particularly relevant where proven interventions remain inaccessible or inconsistently delivered. Conversely, where the barriers and effective delivery strategies are already sufficiently understood, direct investment in services may offer greater benefit than generating additional research. Lower publication volume relative to DALYs should therefore not be interpreted as evidence that research on one disease is inherently insufficient or that publication output should be distributed in exact proportion to disease burden. The appropriate balance among basic, clinical, implementation, and health-systems research depends on the remaining uncertainty and the local barriers to achieving effective care [12,13,14,15,16].

The allocation of resources to research also involves opportunity costs. More healthcare activity is not necessarily beneficial when additional testing, treatment, or system capacity provides little incremental value or diverts resources from more effective care [28,29,30,31]. Health-economic evaluation provides an important complementary perspective because the appropriate allocation of finite resources depends not only on disease burden but also on the costs and expected benefits of alternative actions. Vision impairment itself imposes substantial direct and indirect economic costs [5], while interventions for avoidable vision loss can generate considerable social and economic returns. A systematic review and economic modeling analysis of cataract and refractive-error interventions in low- and middle-income countries reported a median benefit–cost ratio of 36; modeled benefit–cost ratios in India ranged from 28 for vision centers to 42 for school eye screening [32]. These findings illustrate why a high disease burden coupled with low publication output does not establish that the marginal resource should be allocated to additional research. Where important clinical or implementation uncertainties remain, research may generate value by improving effectiveness, efficiency, quality, or equity. Where effective and cost-beneficial interventions are already established and the principal constraint is delivery, direct investment in service coverage, workforce, infrastructure, or treatment access may provide greater benefit than generating additional publications. Conversely, implementation research may itself be economically valuable when resolving uncertainty about how to deliver an intervention more efficiently or equitably. The present bibliometric analysis did not measure costs, health gains, or return on investment and therefore cannot determine which allocation would maximize health or economic benefit in any individual setting.

The positive associations of GDP per capita with both publication volume and citation-weighted impact underscore the importance of the economic and institutional environment in which research is conducted. Countries with greater economic resources may have more developed academic institutions, research funding, trained investigators, bibliographic access, and capacity to publish in highly visible journals. However, the observational design does not establish that greater GDP per capita directly causes greater research output or impact. GDP per capita may also represent other unmeasured characteristics, including national research expenditure, workforce capacity, health-system organization, and access to international collaborations. Commercial support may further influence the volume and composition of ophthalmic research; prior studies have documented industry sponsorship of landmark ophthalmology trials and substantial industry payments related to ophthalmic research [20,21]. Academic incentive structures may also reward publication-based productivity, potentially influencing scholarly output independently of population health need [33]. Neither industry sponsorship nor academic incentive structures were measured in the present analysis, and their contribution to the observed disease- and country-level patterns therefore cannot be determined. Furthermore, the study attributed publications according to author affiliations rather than study location, so the country assigned to a publication does not necessarily represent the population in which the research was conducted or the setting that ultimately benefited from it.

Disease-specific patterns also require contextual interpretation. The high trachoma RBR among high-income countries was strongly influenced by the very small DALY denominator in this income group and should not be interpreted as evidence of uniformly high trachoma research activity across high-income settings. Australia represents an important exception because trachoma remained a domestic public health concern in some Aboriginal and Torres Strait Islander communities during the study period, supporting ongoing surveillance, prevention, and elimination research (Supplement S7) [34]. Australia was subsequently validated by the World Health Organization as having eliminated trachoma as a public health problem in 2026, after the 2011–2021 period examined in this study [35]. Trachoma was also historically endemic in some American Indian communities in the southwestern United States, although this historical experience should not be interpreted as evidence of substantial contemporary endemic burden in the United States [36]. Importantly, our publication-attribution method assigned research according to the corresponding author’s institutional country rather than the location of the population studied. Publications attributed to other high-income countries may therefore represent international collaborations, analyses of populations in endemic countries, laboratory or methodological research, or other contributions to global elimination efforts. The present data cannot distinguish among these mechanisms or infer the motivations underlying such research. Accordingly, the high trachoma RBR in high-income countries reflects both a small burden denominator and heterogeneous research contexts rather than evidence that high-income-country trachoma research uniformly addresses domestic disease or is conducted solely for global public health purposes.

The comparatively limited research output from low- and middle-income countries is particularly relevant because these settings accounted for most of the measured burden from cataract and refractive disorders. Limited research funding, academic infrastructure, mentorship, protected time, and institutional support may constrain locally led research activity [37]. However, publication output is distinct from healthcare delivery, and the type of research needed may differ substantially by setting. Where effective interventions exist but coverage or outcomes remain inadequate, locally led implementation and health-systems research can identify context-specific barriers involving accessibility, affordability, workforce distribution, referral pathways, surgical quality and safety, treatment uptake, follow-up, and equitable delivery [17,25,38]. Such questions are not interchangeable with basic or therapeutic research and may have substantial clinical and public health value even when they generate fewer publications or citations. At the same time, implementation research should not be presumed necessary when the relevant barriers and solutions are already known; in those circumstances, direct investment in proven services may be the more appropriate use of finite resources. Accordingly, greater publication volume should not be assumed to improve access or reduce disparities without corresponding improvements in service delivery and patient outcomes.

The 34 prespecified high-burden, low-publication country–disease pairs provide more specific signals regarding settings that may warrant further assessment (Supplement S10). No corresponding country-attributed publications were identified in the dataset for these pairs, including cataract in Myanmar and AMD in Peru. These findings should be interpreted as bibliometric screening indicators rather than evidence that additional research is necessarily required. Relevant evidence may have been generated elsewhere, published in journals not indexed by Scopus, reported in local languages, disseminated through governmental or nongovernmental reports, or attributed to an internationally based corresponding author. The identified pairs therefore provide a reproducible starting point for consultation with local clinicians, researchers, health authorities, and patient communities to determine whether the principal need is additional research, implementation work, or direct service investment.

This study has several strengths. It integrated disease-burden, bibliographic, economic, and population data over an 11-year period and included 204 countries and territories in the analytic dataset. The distinction between 64,671 globally unique articles and 76,937 country-attributed article–disease records allowed articles relevant to multiple included diseases to contribute appropriately to each disease-specific analysis. Retaining country–disease-year observations with no identified publications also permitted the analysis to capture low-output settings rather than limiting the models to countries already producing research. In addition, the use of both publication volume and citation-weighted impact provided complementary measures of research activity, while the descriptive ratios, correlation analyses, and multivariable models examined the findings from several perspectives.

Several limitations should also be considered. First, Scopus does not capture all journals, local-language publications, gray literature, conference proceedings, policy reports, or other forms of research dissemination. The search restrictions and disease-specific terminology may also have omitted relevant articles or included some records whose primary focus differed from the assigned disease category. Research from lower-resource settings may consequently have been underestimated. Second, assigning each publication to one country based primarily on the corresponding author’s affiliation simplified multinational collaboration and may have underrepresented contributions from coauthors in other countries. It also measured institutional authorship rather than study location. Researchers originating or trained in lower-resource countries but working at institutions elsewhere were attributed to their host institution; author nationality, country of origin, training location, and researcher mobility were not assessed. Third, articles retrieved in more than one disease-specific search were retained once within each applicable disease category. Accordingly, aggregated article–disease records should not be interpreted as the number of globally unique articles. Fourth, citation-weighted impact represents academic citation performance rather than direct clinical, policy, or public health influence. Citation counts were obtained at a single point in time and were not normalized for publication year, disease field, journal, or country, which may disadvantage more recent articles and research from less frequently cited fields. Fifth, the analysis used modeled DALY estimates and did not incorporate uncertainty intervals around disease burden. This uncertainty should also be considered when interpreting differences in cumulative burden among income groups, particularly because the reported income-group shares reflect absolute rather than population- or age-standardized DALYs. Burden-normalized ratios may also be unstable when DALY denominators are small. Sixth, the analysis was limited to five disease categories for which publication searches could be matched to consistently available, disaggregated burden data. The results therefore do not represent the full spectrum of ophthalmic disease, including conditions such as diabetic retinopathy. Seventh, the regression models identify associations and may be affected by residual confounding from factors not measured in the analysis, including research expenditure, number of ophthalmologists and researchers, academic infrastructure, external funding, and international collaboration. The study did not systematically classify individual publications by funding source or industry sponsorship and therefore could not determine the contribution of commercial support to the observed research patterns. Academic promotion criteria and other publication-based incentive structures were also not measured and may influence scholarly output independently of disease burden. Finally, the use of each country’s modal income classification over 2011–2021 may obscure changes in economic classification during the study period. More fundamentally, bibliometric output is not a surrogate for healthcare access or population health improvement. The study did not measure service availability, utilization, treatment coverage, quality of care, affordability, or clinical outcomes and therefore cannot determine whether greater publication output translates into more equitable or effective eye care.

Future studies could incorporate additional bibliographic databases, fractional authorship methods, study-location data, international collaboration networks, and uncertainty in burden estimates. Measures such as clinical trial activity, policy citations, guideline incorporation, service uptake, treatment coverage, research funding, and local research capacity could complement conventional publication and citation metrics. Such analyses would provide a more complete assessment of whether ophthalmology research is translated into improvements in eye health and whether those benefits reach the populations bearing the greatest burden.

## 5. Conclusions

Global ophthalmology research output was unevenly distributed relative to disease burden across disease categories, countries, and income groups from 2011 to 2021. Cataract and refractive disorders accounted for most of the measured disease burden but a smaller proportion of publication volume, while high-income countries produced most publication volume and citation-weighted impact despite accounting for a minority of cumulative DALYs. In the primary population-adjusted models, absolute DALYs were not independently associated with publication volume, whereas GDP per capita and national population were strongly associated with publication volume. DALYs retained a small association with positive citation-weighted impact, while GDP per capita and population remained strongly associated with that outcome.

These findings describe bibliometric patterns rather than an optimal allocation of research resources and should not be interpreted as evidence that increasing publication volume would itself improve access to care or reduce disparities. Research and service-delivery priorities should instead be determined locally by considering disease burden together with remaining uncertainty, effectiveness of existing interventions, implementation barriers, equity, feasibility, expected health benefit, and opportunity cost. In settings where important clinical questions remain unresolved, additional basic or clinical research may be warranted; where effective interventions already exist but coverage or outcomes remain inadequate, implementation and health-systems research may be more relevant; and where the principal barriers and effective solutions are already well understood, direct investment in proven services may offer greater benefit than additional research.

Future studies should move beyond publication and citation metrics by incorporating research type, funding source and industry sponsorship, study location, research expenditure, international collaboration, and local research capacity. Linking these measures with service availability, treatment coverage, affordability, quality of care, cost-effectiveness, and patient outcomes would help determine when and how research activity translates into equitable improvements in population eye health.

## Figures and Tables

**Table 1:** Ophthalmology Research output, citation-weighted impact, and disease burden by modal World Bank income group and disease category, 2011–2021.

Modal Income Group	Disease	Publication Volume, Article–Disease Records	Cumulative Citation-Weighted Impact	Cumulative Disease Burden (DALYs)	RBR per 10,000 DALYs	IBR per 10,000 DALYs
**High Income**	AMD	9501	11,983	1,404,892	68	85
	Cataract	14,471	16,517	5,733,489	25	29
	Glaucoma	16,298	19,052	1,715,321	95	111
	Refractive Disorders	10,795	12,518	8,542,851	13	15
	Trachoma	631	750	4196	1504	1788
**Upper-Middle Income**	AMD	1838	1791	2,153,557	9	8
	Cataract	5966	4467	19,655,914	3	2
	Glaucoma	5254	4161	2,674,635	20	16
	Refractive Disorders	5627	4708	24,558,832	2	2
	Trachoma	96	70	90,593	11	8
**Lower-Middle Income**	AMD	356	339	1,724,077	2	2
	Cataract	2333	2076	37,082,671	1	1
	Glaucoma	1930	1819	2,636,544	7	7
	Refractive Disorders	1362	1280	30,553,906	0	0
	Trachoma	83	77	654,828	1	1
**Low Income**	AMD	14	14	279,566	1	1
	Cataract	128	121	3,677,864	0	0
	Glaucoma	88	82	601,876	1	1
	Refractive Disorders	86	81	3,528,856	0	0
	Trachoma	79	81	648,093	1	1

Data are aggregated over the 2011–2021 study period. Publication volume represents country-attributed article–disease records. Citation-weighted impact was calculated as the sum of log_10_(1 + citation count) across included records. The Research-to-Burden Ratio and Impact-to-Burden Ratio are presented per 10,000 disability-adjusted life years to facilitate comparison across disease and income strata. AMD indicates age-related macular degeneration; DALYs, disability-adjusted life years; IBR, Impact-to-Burden Ratio; and RBR, Research-to-Burden Ratio. Income-group summaries include 76,936 of the 76,937 country-attributed article–disease records; one record lacked an available income-group classification and was excluded from income-group summaries.

**Table 2:** Multivariable associations of disease burden, GDP per capita, national population, disease category, and publication year with ophthalmology publication volume and positive citation-weighted impact, 2011–2021.

Characteristic	Publication Volume Model Adjusted Count Ratio (95% CI)	*p* Value	Positive Citation-Weighted Impact Model Adjusted Ratio of Means (95% CI)	*p* Value
**Main Predictors**				
Annual DALYs, per 1-SD increase	0.99 (0.98 to 1.01)	0.278	1.05 (1.03 to 1.06)	<0.001
GDP per capita, per 1-SD increase	4.47 (4.22 to 4.73)	<0.001	2.86 (2.72 to 3.00)	<0.001
Log population, per 1-SD increase	13.99 (13.02 to 15.03)	<0.001	4.96 (4.63 to 5.32)	<0.001
**Disease Category**				
Glaucoma	2.34 (2.06 to 2.67)	<0.001	1.55 (1.40 to 1.72)	<0.001
Cataract	2.54 (2.24 to 2.89)	<0.001	1.46 (1.32 to 1.61)	<0.001
Refractive Disorders	1.97 (1.72 to 2.25)	<0.001	1.36 (1.22 to 1.51)	<0.001
Trachoma	0.10 (0.08 to 0.12)	<0.001	0.25 (0.20 to 0.30)	<0.001
**Publication Year**				
2012	1.05 (0.84 to 1.30)	0.692	1.00 (0.85 to 1.17)	0.984
2013	1.11 (0.90 to 1.38)	0.323	0.95 (0.81 to 1.12)	0.562
2014	1.06 (0.85 to 1.33)	0.617	0.99 (0.84 to 1.17)	0.912
2015	1.23 (1.00 to 1.52)	<0.05	1.05 (0.90 to 1.23)	0.564
2016	1.43 (1.16 to 1.77)	<0.001	1.23 (1.01 to 1.50)	<0.05
2017	1.47 (1.20 to 1.81)	<0.001	1.05 (0.89 to 1.23)	0.579
2018	1.34 (1.09 to 1.66)	<0.05	0.95 (0.81 to 1.12)	0.536
2019	1.37 (1.11 to 1.68)	<0.05	0.98 (0.84 to 1.15)	0.800
2020	1.70 (1.39 to 2.08)	<0.001	0.99 (0.85 to 1.16)	0.923
2021	1.69 (1.38 to 2.08)	<0.001	0.91 (0.78 to 1.07)	0.259

(a). Negative binomial regression modeled annual country–disease publication volume, and a Gamma generalized linear model with a log link modeled positive annual country–disease citation-weighted impact. Both models included all listed covariates and used HC0 heteroskedasticity-consistent robust standard errors. The publication-volume model included 10,825 country–disease-year observations with complete covariate and population data. The citation-weighted-impact model included 3036 observations with positive citation-weighted impact; 7789 observations with zero citation-weighted impact were excluded from the Gamma model. Fifty-five Taiwan country–disease-year observations were excluded because national population data were unavailable. Exponentiated coefficients are presented as adjusted count ratios for publication volume and adjusted ratios of means for positive citation-weighted impact. No exposure offset was included in the negative binomial model. (b). DALYs, GDP per capita, and natural-log-transformed population were standardized; estimates represent the multiplicative association corresponding to a 1-standard-deviation increase. (c). The reference disease category was age-related macular degeneration. (d). The reference publication year was 2011. Abbreviations: aCR, adjusted count ratio; aRM, adjusted ratio of means; CI, confidence interval; DALYs, disability-adjusted life years; GDP, gross domestic product; SD, standard deviation.

## Data Availability

The disease-burden, economic, and population data supporting this study are publicly available from the Global Burden of Disease database, the World Bank, and the International Monetary Fund. Publication metadata were obtained from Scopus and may be subject to database licensing restrictions. The processed analytic data are available from the corresponding author upon reasonable request, where permitted by licensing restrictions.

## Supplementary Materials

The following supporting information can be downloaded at https://www.mdpi.com/article/10.3390/vision10040061/s1: Supplement S1: Scopus search strategies and raw search record counts; Supplement S2: Ophthalmology Research output and disease burden by modal World Bank income group, 2011–2021; Supplement S3: Top 10 countries by cumulative publication volume, 2011–2021; Supplement S4: Top 10 countries by cumulative citation-weighted impact, 2011–2021; Supplement S5: Geographic distribution of the Impact-to-Burden ratio for age-related macular degeneration, 2011–2021; Supplement S6: Geographic distribution of the Impact-to-Burden ratio for refractive disorders, 2011–2021; Supplement S7: Geographic distribution of the Impact-to-Burden ratio for trachoma, 2011–2021; Supplement S8: Geographic distribution of the Impact-to-Burden ratio for glaucoma, 2011–2021; Supplement S9: Geographic distribution of the Impact-to-Burden ratio for cataract, 2011–2021; Supplement S10: High-burden, low-publication country-disease pairs stratified by modal World Bank income group, 2011–2021; Supplement S11: Comparison of primary population-adjusted models with models omitting national population, 2011–2021.
