# Supplementary material for: Global Ophthalmology Research Output Relative to Disease Burden and National Income, 2011–2021
Source: Vision (Basel). 2026 Sep 1;10(4):61. doi: 10.3390/vision10040061 (PMC13600168; doi:10.3390/vision10040061)
Supplement: Supplementary file 1 [file vision-10-00061-s001.zip › vision-4457561-supplementary.pdf]

Supplement S1: Scopus Search Strategies and Raw Search Record Counts

Supplement S2. Ophthalmology Research Output and Disease Burden by Modal World Bank Income Group, 2011-2021

Supplement S3: Top 10 Countries by Cumulative Publication Volume, 2011-2021

Supplement S4: Top 10 Countries by Cumulative Citation-Weighted Impact, 2011-2021

Supplement S5: Geographic Distribution of the Impact-to-Burden Ratio for Age-Related Macular Degeneration, 2011-2021

Supplement S6: Geographic Distribution of the Impact-to-Burden Ratio for Refractive Disorders, 2011-2021

Supplement S7: Geographic Distribution of the Impact-to-Burden Ratio for Trachoma, 2011-2021

Supplement S8: Geographic Distribution of the Impact-to-Burden Ratio for Glaucoma, 2011-2021

Supplement S9: Geographic Distribution of the Impact-to-Burden Ratio for Cataract, 2011-2021

Supplement S10. High-Burden, Low-Publication Country-Disease Pairs Stratified by Modal World Bank Income Group, 2011-2021

Supplement S11. Comparison of Primary Population-Adjusted Models With Models Omitting National Population, 2011-2021

Supplement S1: Scopus Search Strategies and Raw Search Record Counts

Glaucoma (25,569):
(TITLE-ABS-KEY(glaucoma OR "open angle glaucoma" OR "primary open angle glaucoma" OR "angle closure glaucoma" OR "primary angle closure glaucoma" OR "normal tension glaucoma" OR "low tension glaucoma" OR "ocular hypertension" OR "pigmentary glaucoma" OR "pseudoexfoliation glaucoma" OR "secondary glaucoma" OR "secondary open angle glaucoma" OR "secondary closed angle glaucoma" OR "neovascular glaucoma" OR "congenital glaucoma" OR "juvenile open angle glaucoma" OR JOAG OR "exfoliation glaucoma") OR INDEXTERMS("glaucoma")) AND PUBYEAR > 2010 AND PUBYEAR < 2022 AND (LIMIT-TO (DOCTYPE , "ar")) AND (LIMIT-TO (EXACTKEYWORD , "Human")) AND (LIMIT-TO (SRCTYPE , "j"))

Cataract (25,024):
(TITLE-ABS-KEY (cataract* OR "lens opacity" OR "posterior subcapsular opacity" OR "nuclear sclerosis" OR "posterior subcapsular cataract" OR "congenital cataract" OR "age-related cataract" OR "cataract extraction" OR "cataract surgery" OR "cortical cataract" OR "brunescent cataract" OR "white cataract" OR "sunflower cataract" OR "christmas tree cataract" OR "radiation cataract" OR "traumatic cataract") OR INDEXTERMS("cataract"/exp)) AND PUBYEAR > 2010 AND PUBYEAR < 2022 AND (LIMIT-TO (SRCTYPE , "j")) AND (LIMIT-TO (DOCTYPE , "ar")) AND (LIMIT-TO (EXACTKEYWORD , "Human"))

Age-Related Macular Degeneration (AMD) (12,708):
(TITLE-ABS-KEY ("age-related macular degeneration" OR "age related macular degeneration" OR (AMD W/3 (macula* OR degenerat*)) OR "wet AMD" OR "dry AMD" OR "neovascular AMD" OR "geographic atrophy" OR "macular degeneration" OR ARMD OR "exudative AMD" OR "exudative macular degeneration" OR "non-exudative AMD" OR "non-exudative macular degeneration") OR INDEXTERMS ("age-related macular degeneration")) AND PUBYEAR > 2010 AND PUBYEAR < 2022 AND (LIMIT-TO (DOCTYPE , "ar")) AND (LIMIT-TO (EXACTKEYWORD , "Human")) AND (LIMIT-TO (SRCTYPE , "j"))

Trachoma (973):
(TITLE-ABS-KEY (trachoma OR "trachomatous trichiasis" OR "trachomatous inflammation" OR ("Chlamydia trachomatis" W/5 (ocular OR eye OR conjunctiva OR eyelid))) OR INDEXTERMS ("trachoma")) AND PUBYEAR > 2010 AND PUBYEAR < 2022 AND (LIMIT-TO (DOCTYPE , "ar")) AND (LIMIT-TO (EXACTKEYWORD , "Human")) AND (LIMIT-TO (SRCTYPE , "j"))

Refractive Disorders/Errors (19,217):
(TITLE-ABS-KEY ("refractive error*" OR "refractive disorder*" OR myopia OR hyperopia OR hypermetrop* OR astigmatis* OR presbyopia OR ametropia) OR INDEXTERMS ("refractive error")) AND PUBYEAR > 2010 AND PUBYEAR < 2022 AND (LIMIT-TO (DOCTYPE , "ar")) AND (LIMIT-TO (EXACTKEYWORD , "Human")) AND (LIMIT-TO (SRCTYPE , "j"))

Counts represent raw records returned by each disease-specific Scopus search before within-disease deduplication by Scopus electronic identifier (EID). Across the five searches, 83,491 raw records were retrieved. After duplicate EIDs were removed within each disease category, 79,124 article-disease records remained. Articles retrieved by more than one disease-specific search were retained once within each applicable disease category. Of these records, 76,937 had an assignable country of origin.

Supplement S2. Ophthalmology Research Output and Disease Burden by Modal World Bank Income Group, 2011-2021

Income Group	Cumulative DALYs (n)	Share of Cumulative Disease Burden, %	Publication Volume, Article-Disease Records	Share of Publication Volume, %	Cumulative Citation-Weighted Impact	Share of Citation-Weighted Impact, %
High Income	17,400,749	11.8%	51,696	67.2%	60,820	74.2%
Upper-Middle Income	49,133,531	33.2%	18,781	24.4%	15,197	18.5%
Lower-Middle Income	72,652,026	49.1%	6,064	7.9%	5,591	6.8%
Low Income	8,736,255	5.9%	395	0.5%	379	0.5%

Each country or territory was classified according to its modal World Bank income group during 2011-2021. Publication volume was defined as the number of country-attributed article-disease records. Citation-weighted impact was calculated by summing $\log_{10}(1 + \text{citation count})$ across article-disease records. DALYs indicates disability-adjusted life years. Income-group summaries include 76,936 of the 76,937 country-attributed article-disease records; one record lacked an available income-group classification and was excluded from income-group summaries.

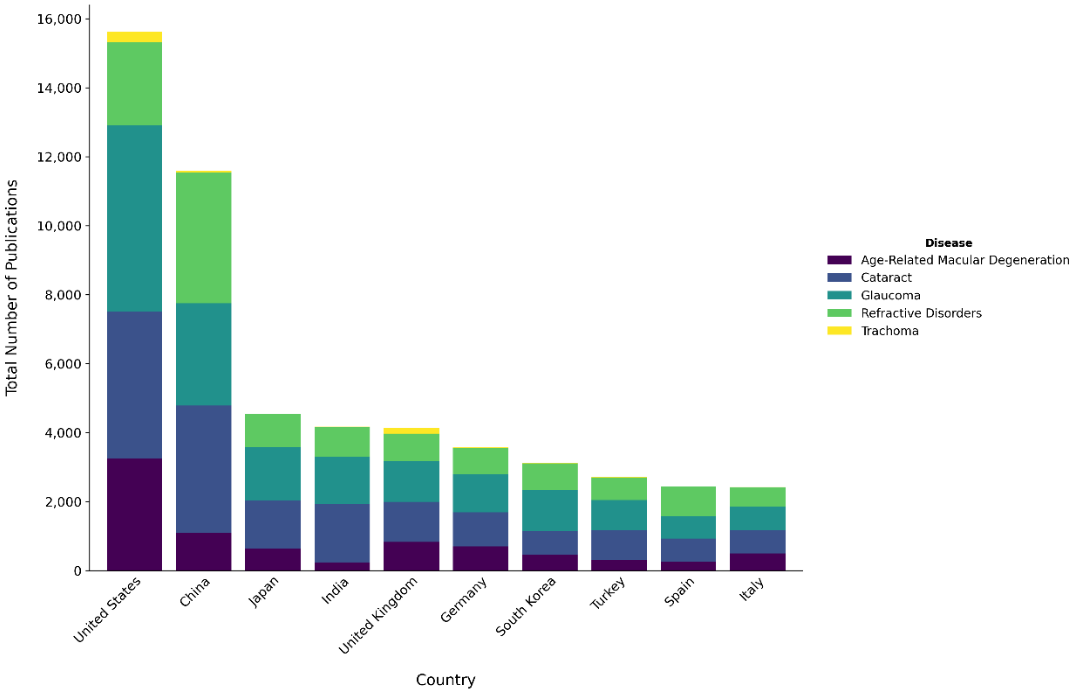

Supplement S3: Top 10 Countries by Cumulative Publication Volume, 2011-2021. The stacked bar chart shows the 10 countries with the greatest cumulative publication volume during 2011-2021. Publication volume was defined as the number of country-attributed article-disease records. Each bar represents one country, and its total height indicates cumulative publication volume. Colored segments show the contribution of each disease category to the country's publication portfolio. Because articles relevant to more than one included disease could contribute to each applicable disease category, these totals should not be interpreted as numbers of globally unique articles. The figure was generated using Python 3.12.4 with Matplotlib.

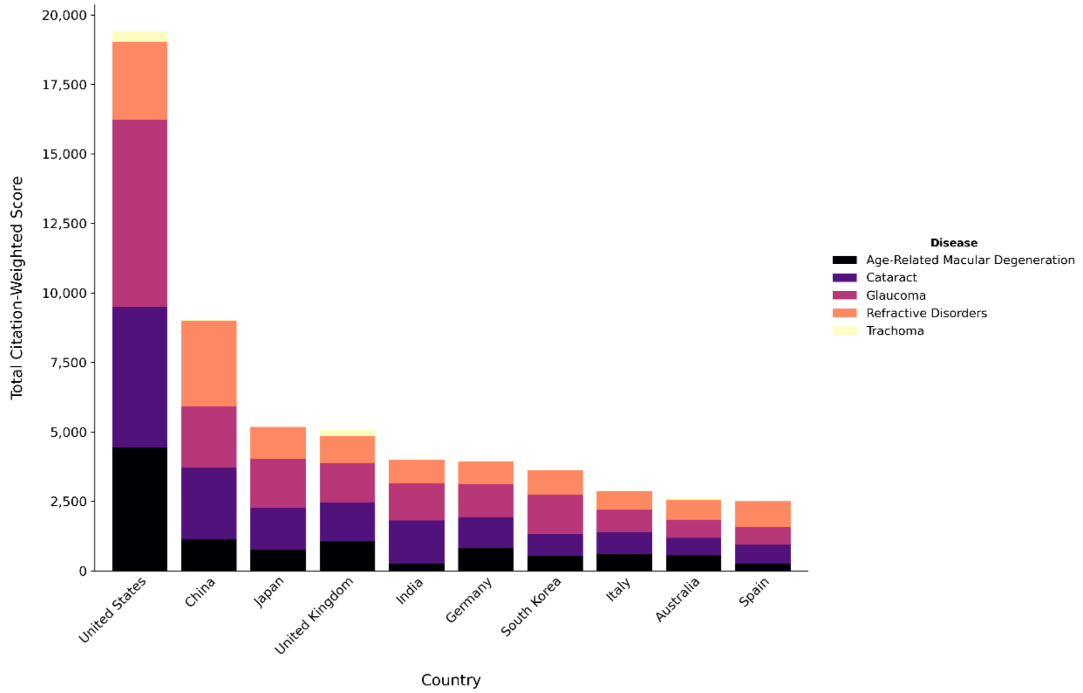

Supplement S4: Top 10 Countries by Cumulative Citation-Weighted Impact, 2011-2021. The stacked bar chart shows the 10 countries with the greatest cumulative citation-weighted impact during 2011-2021. Citation-weighted impact was calculated by summing $\log_{10}(1 + \text{citation count})$ across country-attributed article-disease records. Each bar represents one country, and its total height indicates cumulative citation-weighted impact. Colored segments show the contribution of each disease category to the country's citation-weighted impact portfolio. The figure was generated using Python 3.12.4 with Matplotlib.

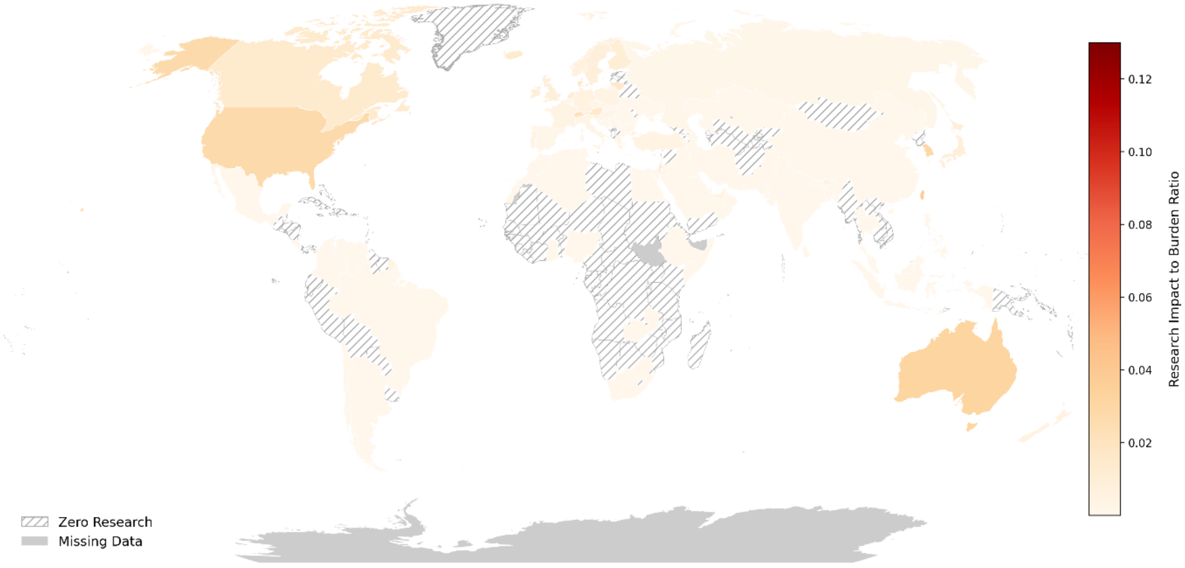

Supplement S5: Geographic Distribution of the Impact-to-Burden Ratio for Age-Related Macular Degeneration, 2011-2021. The map shows the country-level Impact-to-Burden Ratio (IBR) for age-related macular degeneration, calculated as cumulative citation-weighted impact divided by cumulative disability-adjusted life years (DALYs) for that disease during 2011-2021. Citation-weighted impact was calculated by summing $\log_{10}(1 + \text{citation count})$ across country-attributed article-disease records. Darker shading indicates greater citation-weighted impact per unit of disease burden. Diagonal hatching, labeled "Zero Research" or "Zero Research Impact" in the map key, indicates zero citation-weighted impact in the dataset. Gray shading indicates missing data. The color scale is specific to this disease and should not be compared directly with the color scales of the other disease-specific maps. The map was generated using Python 3.12.4 with GeoPandas and Matplotlib.

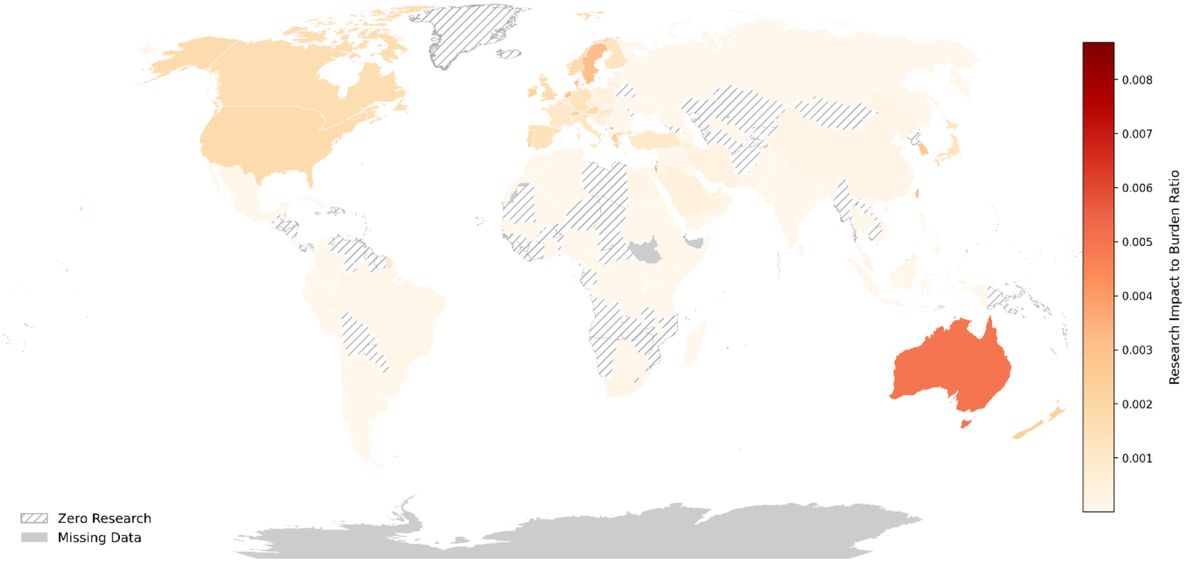

Supplement S6: Geographic Distribution of the Impact-to-Burden Ratio for Refractive Disorders, 2011-2021. The map shows the country-level Impact-to-Burden Ratio (IBR) for refractive disorders, calculated as cumulative citation-weighted impact divided by cumulative disability-adjusted life years (DALYs) for that disease during 2011-2021. Citation-weighted impact was calculated by summing $\log_{10}(1 + \text{citation count})$ across country-attributed article-disease records. Darker shading indicates greater citation-weighted impact per unit of disease burden. Diagonal hatching, labeled "Zero Research" or "Zero Research Impact" in the map key, indicates zero citation-weighted impact in the dataset. Gray shading indicates missing data. The color scale is specific to this disease and should not be compared directly with the color scales of the other disease-specific maps. The map was generated using Python 3.12.4 with GeoPandas and Matplotlib.

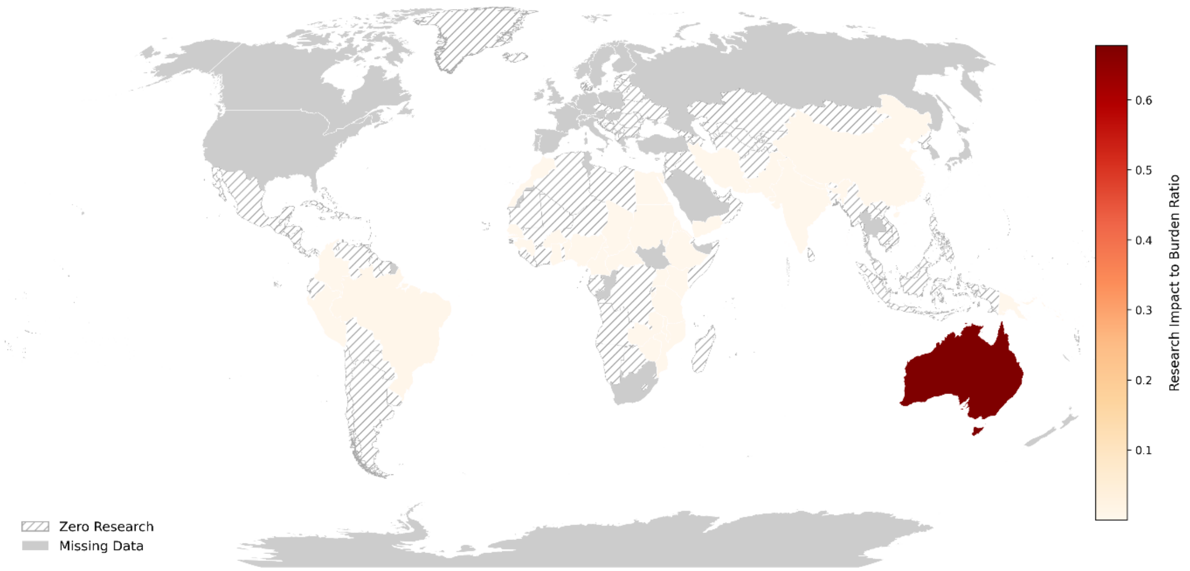

Supplement S7: Geographic Distribution of the Impact-to-Burden Ratio for Trachoma, 2011-2021. The map shows the country-level Impact-to-Burden Ratio (IBR) for trachoma, calculated as cumulative citation-weighted impact divided by cumulative disability-adjusted life years (DALYs) for that disease during 2011-2021. Citation-weighted impact was calculated by summing $\log_{10}(1 + \text{citation count})$ across country-attributed article-disease records. Darker shading indicates greater citation-weighted impact per unit of disease burden. Diagonal hatching, labeled "Zero Research" or "Zero Research Impact" in the map key, indicates zero citation-weighted impact in the dataset. Gray shading indicates missing data. The color scale is specific to this disease and should not be compared directly with the color scales of the other disease-specific maps. The map was generated using Python 3.12.4 with GeoPandas and Matplotlib.

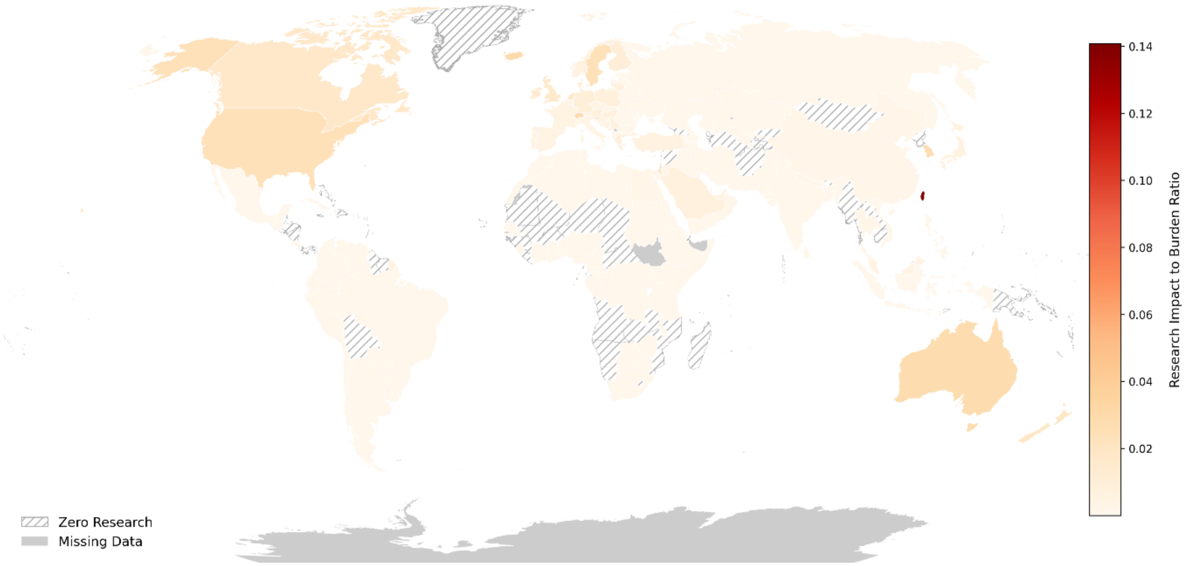

Supplement S8: Geographic Distribution of the Impact-to-Burden Ratio for Glaucoma, 2011-2021. The map shows the country-level Impact-to-Burden Ratio (IBR) for glaucoma, calculated as cumulative citation-weighted impact divided by cumulative disability-adjusted life years (DALYs) for that disease during 2011-2021. Citation-weighted impact was calculated by summing $\log_{10}(1 + \text{citation count})$ across country-attributed article-disease records. Darker shading indicates greater citation-weighted impact per unit of disease burden. Diagonal hatching, labeled "Zero Research" or "Zero Research Impact" in the map key, indicates zero citation-weighted impact in the dataset. Gray shading indicates missing data. The color scale is specific to this disease and should not be compared directly with the color scales of the other disease-specific maps. The map was generated using Python 3.12.4 with GeoPandas and Matplotlib.

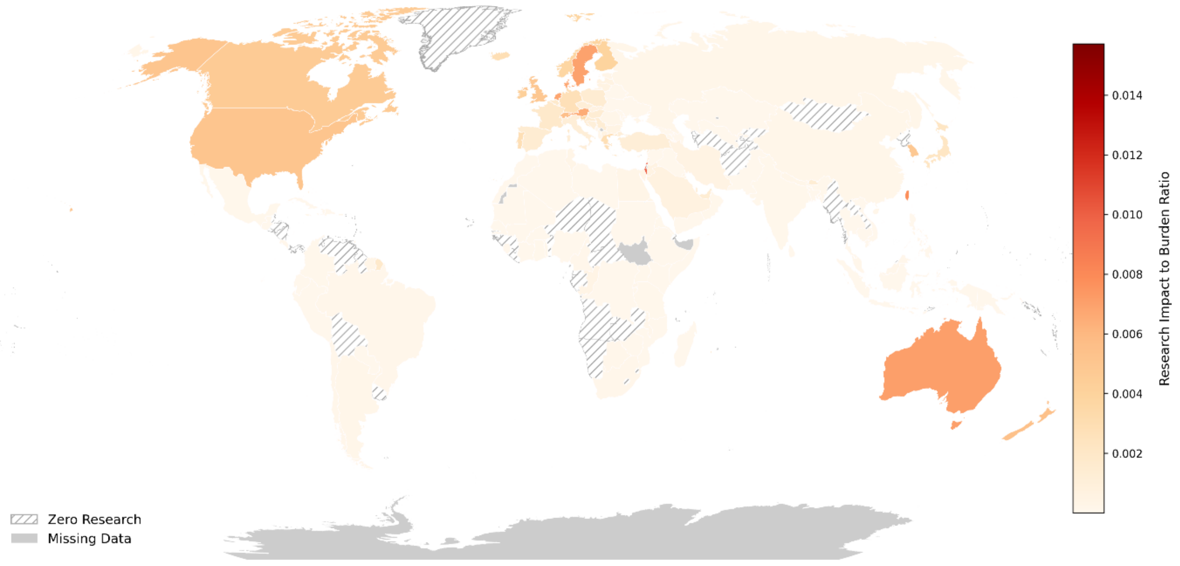

Supplement S9: Geographic Distribution of the Impact-to-Burden Ratio for Cataract, 2011-2021. The map shows the country-level Impact-to-Burden Ratio (IBR) for cataract, calculated as cumulative citation-weighted impact divided by cumulative disability-adjusted life years (DALYs) for that disease during 2011-2021. Citation-weighted impact was calculated by summing $\log_{10}(1 + \text{citation count})$ across country-attributed article-disease records. Darker shading indicates greater citation-weighted impact per unit of disease burden. Diagonal hatching, labeled "Zero Research" or "Zero Research Impact" in the map key, indicates zero citation-weighted impact in the dataset. Gray shading indicates missing data. The color scale is specific to this disease and should not be compared directly with the color scales of the other disease-specific maps. The map was generated using Python 3.12.4 with GeoPandas and Matplotlib.

Supplement S10. High-Burden, Low-Publication Country-Disease Pairs Stratified by Modal World Bank Income Group, 2011-2021				
**Country**	**Disease**	**Income Group**	**Cumulative DALYs**	**Publication Volume, Article-Disease Records**
**High Income**				
Oman	Trachoma	High Income	4,130.95	0
Nauru	Trachoma	High Income	0.71	0
**Upper-Middle Income**				
Peru	AMD	Upper-Middle Income	36,594.52	0
Algeria	Trachoma	Upper-Middle Income	6,305.73	0
Iraq	Trachoma	Upper-Middle Income	4,207.28	0
Mexico	Trachoma	Upper-Middle Income	3,863.11	0
Libya	Trachoma	Upper-Middle Income	757.14	0
Fiji	Trachoma	Upper-Middle Income	78.94	0
American Samoa	Trachoma	Upper-Middle Income	2.77	0
**Lower-Middle Income**				
Myanmar	Cataract	Lower-Middle Income	913,722.47	0
Myanmar	Refractive Disorders	Lower-Middle Income	520,259.18	0
Vietnam	AMD	Lower-Middle Income	61,836.86	0
Kenya	AMD	Lower-Middle Income	29,878.21	0
Vietnam	Trachoma	Lower-Middle Income	24,001.06	0
Sudan	AMD	Lower-Middle Income	21,598.39	0
Myanmar	AMD	Lower-Middle Income	18,863.94	0
Cote d'Ivoire	Trachoma	Lower-Middle Income	9,766.44	0
Myanmar	Trachoma	Lower-Middle Income	6,346.88	0
**Low Income**				
Afghanistan	Refractive Disorders	Low Income	228,832.99	0
Afghanistan	Cataract	Low Income	196,697.74	0
North Korea	Refractive Disorders	Low Income	138,286.79	0
Niger	Cataract	Low Income	89,693.14	0
Somalia	Trachoma	Low Income	71,953.26	0
Niger	Trachoma	Low Income	31,473.10	0
Afghanistan	Glaucoma	Low Income	29,397.91	0
Tanzania	AMD	Low Income	27,182.76	0
Mali	Glaucoma	Low Income	24,020.71	0
Niger	Glaucoma	Low Income	23,984.35	0
Afghanistan	AMD	Low Income	16,618.06	0
Mali	Trachoma	Low Income	13,268.59	0
Mozambique	AMD	Low Income	12,595.68	0
Uganda	AMD	Low Income	8,066.80	0
Niger	AMD	Low Income	7,511.82	0
Malawi	AMD	Low Income	7,300.92	0

This table lists the 34 country-disease pairs that met the prespecified high-burden, low-publication definition. Each country or territory was assigned its modal World Bank income classification during 2011-2021. Within each disease and modal income-group stratum, a high-burden, low-publication pair was defined as cumulative disease burden at or above the 75th percentile and publication volume at or below the 25th percentile. Publication volume was defined as the number of country-attributed article-disease records. In every stratum in which a pair was identified, the 25th-percentile publication-volume threshold was zero; therefore, all listed country-disease pairs had no attributed article-disease records. This designation is descriptive and should not be interpreted as evidence that additional research is necessarily preferable to implementation or direct service investment. Because burden ranking was performed within each disease and modal income-group stratum, “high burden” is relative to that stratum rather than an absolute

burden threshold. Accordingly, pairs may meet the definition in strata where absolute disease burden is uniformly very low, as illustrated by trachoma in Nauru (0.71 cumulative DALYs) and American Samoa (2.77 cumulative DALYs); such pairs should not be interpreted as substantive evidence of a large unmet research need. Abbreviations: AMD, age-related macular degeneration; DALYs, disability-adjusted life years.

**Supplement S11. Comparison of Primary Population-Adjusted Models With Models Omitting National Population, 2011-2021**			
**Outcome**	**Predictor**	**Model Omitting Population**	**Primary Population-Adjusted Model**
Publication volume	Annual DALYs, per 1-SD increase	aCR, 27.68 (15.66 to 48.92); p < 0.001	aCR, 0.99 (0.98 to 1.01); p = 0.278
Publication volume	GDP per capita, per 1-SD increase	aCR, 5.07 (4.72 to 5.45); p < 0.001	aCR, 4.47 (4.22 to 4.73); p < 0.001
Positive citation-weighted impact	Annual DALYs, per 1-SD increase	aRM, 1.61 (1.32 to 1.97); p < 0.001	aRM, 1.05 (1.03 to 1.06); p < 0.001
Positive citation-weighted impact	GDP per capita, per 1-SD increase	aRM, 2.69 (2.49 to 2.91); p < 0.001	aRM, 2.86 (2.72 to 3.00); p < 0.001
For each outcome, the models with and without population were fitted on the same outcome-specific population-complete analytic sample. The primary models additionally included natural-log-transformed annual national population. In the primary models, a 1-standard-deviation increase in log population was associated with publication volume (aCR, 13.99; 95% CI, 13.02-15.03; p < 0.001) and positive citation-weighted impact (aRM, 4.96; 95% CI, 4.63-5.32; p < 0.001). Variance inflation factors were below 1.4 in both analytic samples, providing no evidence of problematic multicollinearity. The publication-volume models included 10,825 country-disease-year observations. The Gamma models included 3,036 observations with positive citation-weighted impact; 7,789 observations with zero citation-weighted impact were excluded. Fifty-five Taiwan country-disease-year observations were excluded because national population data were unavailable. Abbreviations: aCR, adjusted count ratio; aRM, adjusted ratio of means; CI, confidence interval; DALYs, disability-adjusted life years; GDP, gross domestic product; SD, standard deviation.			
